# Traditional Medicinal Ranunculaceae Species from Romania and Their In Vitro Antioxidant, Antiproliferative, and Antiparasitic Potential

**DOI:** 10.3390/ijms252010987

**Published:** 2024-10-12

**Authors:** Cristina D. Heller, Farnaz Zahedifard, Ivo Doskocil, Doru Pamfil, Martin Zoltner, Ladislav Kokoska, Johana Rondevaldova

**Affiliations:** 1 Laboratory of Molecular Therapy, Institute of Biotechnology of the Czech Academy of Sciences, Prumyslova 595, 252 50 Vestec, Czech Republic; cristinadaniela.heller@ibt.cas.cz; 2 Department of Parasitology, Faculty of Science, Charles University, 252 50 Prague, Czech Republic; zahediff@natur.cuni.cz (F.Z.); zoltnerm@natur.cuni.cz (M.Z.); 3 Department of Microbiology, Nutrition and Dietetics, Faculty of Agrobiology, Food and Natural Resources, Czech University of Life Sciences Prague, Kamycka 129, 165 00 Prague-Suchdol, Czech Republic; doskocil@af.czu.cz; 4 Department of Horticulture and Landscape Architecture, Faculty of Horticulture, University of Agricultural Sciences and Veterinary Medicine, 3-5 Mănăştur Street, 400372 Cluj-Napoca, Romania; dpamfil@gmail.com; 5 Department of Crop Sciences and Agroforestry, Faculty of Tropical AgriSciences, Czech University of Life Sciences Prague, Kamycka 129, 165 00 Prague-Suchdol, Czech Republic; kokoska@ftz.czu.cz

**Keywords:** antileishmanial, antioxidants, antitrypanosomal, buttercup family, cytotoxicity, medicinal plants, plant extract

## Abstract

Several Ranunculaceae species are used in folk medicine to eliminate pathologies associated with oxidative stress as well as parasitic infections; however, a number of studies confirming their pharmacological properties is limited. In this study, 19 ethanolic extracts obtained from 16 Ranunculaceae species were assayed for in vitro antioxidant, antiproliferative, and antiparasitic potential. The maximum antioxidant potential in both oxygen radical absorbance capacity (ORAC) and 2,2-diphenyl-1-picrylhydrazyl (DPPH) assays was observed for *Aconitum toxicum* extract [half-maximal inhibitory concentration (IC_50_) 18.7 and 92.6 μg/mL]. Likewise, *Anemone transsilvanica* extract exerted the most promising antiproliferative activity against Caco-2 (IC_50_ 46.9 μg/mL) and HT29 (IC_50_ 70.2 μg/mL) cell lines in 3-(4,5-dimethylthiazol-2-yl)-2,5-diphenyltetrazolium bromide (MTT) assay. Additionally, a dual antioxidant and cytotoxicity effect was demonstrated for *Aconitum moldavicum* and *Caltha palustris* extracts. Whilst the efficacy of extracts was modest against *Trypanosoma brucei* (IC_50_ ranging from 88.8 to 269.3 µg/mL), several extracts exhibited high potency against *Leishmania infantum* promastigotes (*Aconitum vulparia* IC_50_ 18.8 µg/mL). We also tested them against the clinically relevant intracellular stage and found extract of *A. vulparia* to be the most effective (IC_50_ 29.0 ± 1.1 µg/mL). All tested extracts showed no or low toxicity against FHs 74Int normal cell line (IC_50_ ranging from 152.9 to >512 µg/mL). In conclusion, we suggest the above-mentioned plant extracts as potential candidates for development of novel plant-based antioxidant and/or antiproliferative and/or antileishmanial compounds.

## 1. Introduction

An interest in plants with medical potential and pharmaceutical usage has risen dramatically in recent years due to their diverse bioactive properties, which include anticancer, antioxidant, and antiparasitic effects. About 1300 medicinal plants are used in Europe, of which 90% are harvested from wild resources [1]. Ranunculaceae (buttercup family) is one of the largest flowering plant families comprising about 60 genera and 2500 species distributed worldwide, with most found in the temperate and cold areas of the northern hemisphere [2]. In Romania, the family is represented by 23 genera and approximately 110 species including rare and endemic plants, distributed in all regions of the country [3]. Although many species belonging to the family are well-known for being highly poisonous, a number of them have been used for centuries as medicines, spices, and vegetables after cautious processing to reduce their toxicity [4]. In folk medicine, Ranunculaceae species have been used in heat-clearing, detoxification, and as a natural remedy for malaria and various ailments related to oxidative stress such as asthma, arthritis, bronchitis, cancer, gout, and rheumatism [5,6,7]. At present, a broad spectrum of pharmacological activities, including anti-inflammatory, analgesic, antimicrobial, antiparasitic, and antitumor properties have been reported for various Ranunculaceae species [4]. Currently, a number of derivatives of compounds isolated from species belonging to the family (e.g., cimifugaside, hellebrigenin, hydrastine, and thymoquinone) are used as treatment for several conditions, such as cancer, cardiac dysfunctions, and various types of inflammation [8,9,10]. 

It has been observed that many members of the family exhibit a strong free radical scavenging activity on top of the various secondary metabolites (e.g., diterpenoid alkaloids, triterpenoid saponins, thymoquinone) isolated from *Aconitum*, *Anemone*, *Consolida*, *Delphinium,* and *Nigella* species. Moreover, they are also described as one of the most promising natural compounds for treating multiple types of cancer (e.g., breast cancer, colon cancer, epidermoid carcinoma, gastric carcinoma, and prostate cancer among others) via modulating multiple signal pathways involved in cancer initiation and progression [4,11,12,13,14,15,16,17]. Several studies of crude extracts and isolated compounds of *Consolida* species support its utilization as anthelmintic herbals and reveal their high potential as treatment options for protozoal infections, in particular against *Leishmania* sp., *Plasmodium* sp., and *Trypanosoma* spp. [18,19]. A set of flavonol glycosides, bisbenzyl isoquinoline alkaloids, and diterpenoid alkaloids such as 15,22-O-diacetyl-19-oxo-dihydroatisine, azitine, and isoazitine obtained from *Aconitum*, *Delphinium*, and *Consolida* species displayed promising antileishmanial and antitrypanocidal properties [18,20,21]. Although the high potential of Ranunculaceae species as medicinal plants has been recognized, many of the family members, including endemic plants used in folk medicine, remain uncharacterized regarding their biological activities.

Thus, the main aim of this study was to investigate 19 ethanolic extracts obtained from 16 species belonging to the Ranunculaceae family, namely, *Aconitum moldavicum* Hacq., *A. toxicum* Rchb, *A. variegatum* L., *A. vulparia* Rchb., *Anemone transsilvanica* (Fuss) Heuff., *Caltha palustris* L., *Hepatica nobilis* Mill., *Ranunculus acris* L., *R. bulbosus* L., *R. carpaticus* Herbich, *R. platanifolius* L., *R. polyanthemos* L., *R. repens* L., *R. sardous* Crantz, *R. serpens* subsp. *nemorosus* (DC.) G. López, and *Trollius europaeus* L. for their antioxidant, cytotoxicity, and antiprotozoal effects in vitro. 

## 2. Results and Discussion

In this study, 19 crude extracts of 16 Ranunculaceae species used in folk medicine (Table 1) were assayed for their in vitro antioxidant, antiproliferative and antiprotozoal activities. The complete results of 2,2-diphenyl-1-picrylhydrazyl (DPPH), oxygen radical absorbance capacity (ORAC), 3-(4,5-dimethylthiazol-2-yl)-2,5-diphenyltetrazolium bromide (MTT), and resazurin assays of tested plant extracts are summarized in Table 2.

### 2.1. Antioxidant Effect

Oxidative stress is caused by an imbalance between production and accumulation of reactive oxygen species (ROS) that participate in cell-signaling pathways controlling programmed cell death, gene expression, and mechanisms of immune defense and play a critical role in maintaining homeostasis in living organisms [22,23]. Excessive ROS accumulation in the cells/tissues leads to their damage (oxidative stress), and can be responsible, to varying degrees, for the development of various human non-communicable diseases (NCDs) such as cancer, diabetes, metabolic disorders, atherosclerosis, and cardiovascular diseases [24,25]. Considerable evidence shows that numerous medicinal and edible plant species and various compounds they produce, mainly phenolics, are potent scavengers of ROS [26,27,28]. Furthermore, many species in the Ranunculaceae family are rich in bioactive compounds which help scavenge free radicals and reduce oxidative stress in cells. The most notable of these include flavonoids, phenolic acids, alkaloids, saponins, and tannins [29,30,31,32,33].

In the present study, the most promising free radical scavenging potential has been observed for *A*. *toxicum* (herb) extract in both assays, the ORAC with half-maximal inhibitory concentration (IC_50_) = 18.7 ± 6.6 μg/mL, and the DPPH IC_50_ = 92.6 ± 16.6 μg/mL. Furthermore, based on the results of the ORAC test, *A*. *toxicum* extract showed higher antioxidant potential than positive control Trolox (IC_50_ = 22.4 ± 7.3 μg/mL). Additionally, *A*. *toxicum* extract exhibited relatively low toxicity toward the FHs 74Int human epithelial cell line with an IC_50_ of 274.9 μg/mL. Likewise, significant antioxidant activity was detected for *A*. *moldavicum* (IC_50_ = 23.1 ± 4.6 μg/mL) and *A*. *variegatum* (IC_50_ = 26.4 ± 7.7 μg/mL) extracts in the ORAC assay. The promising antioxidant potential of several *Aconitum* species (including *A*. *toxicum*, *A*. *variegatum*, and *A*. *vulparia*) has been already described [26,31,32,33,34,35]; however, to the best of our knowledge, this is the first report concerning the antioxidant activity of *A*. *moldavicum*. According to the previous reports, the flavonol glycosides are described as the major groups of natural compounds responsible for the antioxidant ability of *Aconitum* species [32,33]. Previously, two flavonol glycosides isolated from methanolic extracts of *Aconitum burnatii* demonstrated scavenging abilities comparable to those of vitamin C and Trolox [36]. Additionally, the same study characterized three new flavonol glycosides from the methanolic extracts of *Aconitum variegatum* (herb) [35]. In a similar study using bioassay-guided fractionation on several methanolic extracts of Italian *Aconitum* species (*A*. *napellus* subsp. *tauricum*, *A*. *napellus* subsp. *neomontanum*, *A*. *paniculatum*, *A*. *vulparia*), 13 flavonol glycosides were isolated, with two fractions revealing the highest activity in the DPPH assay, showing IC_50_ values of 2.0 μg/mL and 2.6 μg/mL, respectively [33]. Furthermore, the phytochemical study of *Aconitum anthora* identified four flavonol glycosides, two of which were isolated for the first time [31]. Similar 3,7-O-glycosides were identified in *Aconitum chiisanense* Nakai [37], *A. napellus* ssp. *tauricum* (Wulfer) Gayer [32], and *A*. *napellus* ssp. *neomontanum* (Wulfer) Gayer [38]. The comparison of data from previous work on the chemical profile of *Aconitum* species has shown that the highest antioxidant potential could be attributed to several flavonols isolated from *A*. *napellus* subspecies. Furthermore, comparing our data with the aforementioned studies suggests that flavonol glycosides contribute to the antioxidant potential of *A*. *toxicum*, *A*. *moldavicum*, and *A*. *variegatum* extracts, although this hypothesis should be verified through further chemical analysis of the extracts examined in this investigation.

In addition, based on the results of the ORAC assay in the present study, the extracts of *Anemone transsilvanica* (leaves), *C*. *palustris*, *H*. *nobilis*(herb), *R*. *acris*, *R*. *platanifolis*, *R*. *repens*, *R*. *serpens* subsp. *nemorosus*, and *T*. *europaeus* exhibited interesting free radical scavenging ability with IC_50_ values in the range from 35.7 to 47.7 μg/mL. The rest of the tested plant extracts exerted medium to weak inhibition of AAPH in the range from 56.4 to 238.6 μg/mL. In the DPPH assay, all extracts (except *A*. *toxicum*) showed only weak (IC_50_ in the range from 104.0 to 240.5 μg/mL) or no antioxidant potential (IC_50_ > 256 μg/mL). These results are in accordance with the findings of other authors, who previously reported the antioxidant activity of *C*. *palustris*, *R*. *acris*, *R. bulbosus*, *R*. *sardous*, and *T*. *europaeus* [34,35,39]. To the best of our knowledge, the antioxidant potential of *A*. *transsilvanica*, *H. nobilis*, *R*. *carpaticus*, *R*. *platanifolius*, *R*. *polyanthemos*, *R*. *repens,* and *R*. *serpens* was evaluated for the first time in present study.

Previously, various triterpenoids isolated from *Anemone cathayensis* demonstrated radical scavenging activity in vitro comparable to that of natural antioxidants (65.9–78.3% at concentrations of 0.05–0.1 mg/mL) [17]. Furthermore, over the last few decades, various studies have investigated the chemical components and pharmacological activities of the *Ranunculus* species [29]. Consequently, the phytochemical analysis of several related *Ranunculus* species has already been described. The phytochemical screening of *R. arvensis* [40], *R. japonicus* [41], and *R. ternatus* [42,43] revealed that these species are rich in glycoside ranunculin, protoanemonin, flavonoids, and saponins.

Finally, various studies suggest that species belonging to the *Ranunculus* genus (e.g., *R. arvensis*, *R. marginatus* var. *trachycarpus*, *R. sprunerianus* Boiss) exhibit potent antioxidant activity, largely due to the presence of bioactive compounds such as flavonoids and polyphenols [13,44]. However, in a previous report by Neag et al. [39] on extracts of *R. ficaria*, *R. bulbosus*, *R. sardous*, and *R. sceleratus*, no direct or linear correlation was found between the content of polyphenols (flavonoids and phenolic acids) and the obtained antioxidant capacity, suggesting that other classes of bioactive compounds may also contribute to the antioxidant effect of the *Ranunculus* species. 

Moreover, we should keep in mind that the choice of solvent used for extraction [45] and seasonal variations in climate directly affect the growth cycle of plants and the concentration of bioactive compounds [46]. In fact, the timing of collection (winter and spring) has been shown to play a major role in the variability of fractions/extracts and consequently is likely to influence their therapeutic efficacy and bioactive potential. For example, Hrichi et al. [47] demonstrated that winter leaves of *Convolvulus althaeoides* L. contained the highest amounts of polyphenolic compounds, while spring-collected leaves harbored the highest pigment content. Therefore, many factors must be considered when comparing the antioxidant activity and chemical composition of various plant extracts.

### 2.2. Anticancer Activity

The anticancer activity of plants has been a major focus of research, as many medicinal plants contain bioactive compounds that can inhibit the growth of cancer cells, induce apoptosis, or affect cancer pathways. The anticancer activity of plant extracts typically refers to the ability of the extract or an isolated compound to inhibit tumor cell proliferation or induce cell death [11]. Previous studies, as reviewed by Hao et al. [11], have revealed that at least 17 genera of the Ranunculaceae (buttercup family) are enriched with anticancer phytometabolites, including alkaloids, terpenoids, saponins, and polysaccharides [4,11].

**Figure 1:**
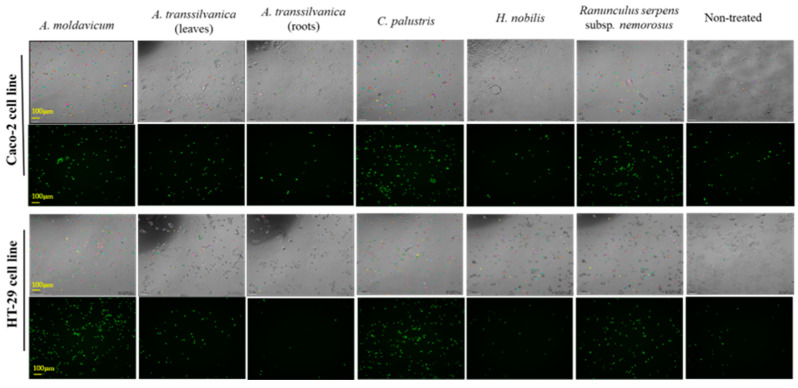
Brightfield and fluorescent image capture (100×) of Caco-2 and HT29 cell lines exposed for 72 h at *A*. *moldavicum*, *A*. *transsilvanica* (leaves and roots extracts), *C*. *palustris*, *H*. *nobilis*, and *R*. *serpens* subsp. *nemorosus* extracts (100 μg/mL) using Lumascope 720 (Etaluma, Carlsbad, CA, USA). The Green dead cell stain SYTOX (15 nM/well) was added together with the extracts to each well and automated brightfield and fluorescent image capture was performed every 2 h.

**Figure 2:**
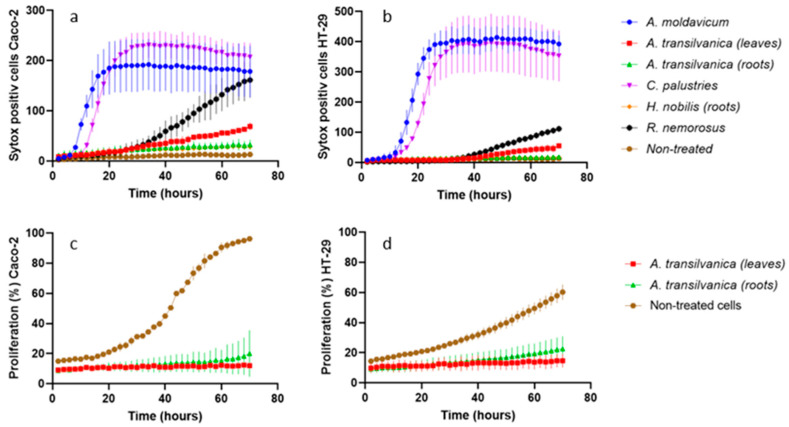
Numbers of SYTOX Green positive Caco-2 (**a**) and HT29 (**b**) cells were quantified based on fluorescent image captures using Lumaquant 8.8 software. The percent of proliferation (%) of the Caco-2 (**c**) and HT29 (**d**) cell lines was calculated based on percentage area of cells over time from brightfield image captures using Lumaquant 8.8 software. The adenocarcinoma cell lines were treated with *A. moldavicum*, *A. transsilvanica* (leaves and roots extracts), *C. palustris*, *H. nobilis*, and *R. serpens* subsp. *nemorosus* ethanolic extracts in a concentration of 100 μg/mL for 72 h. The results are expressed as mean (n = 3) +/− SD.

Based on the MTT assay performed in this study, the extracts obtained from leaves and roots of *A*. *transsilvanica* demonstrated the most promising antiproliferative effect to both Caco-2 (IC_50_ = 46.9 ± 5.9 and 65.8 ± 2.6 μg/mL) and HT29 (IC_50_ = 86.5 ± 4.5 and 70.2 ± 9.4 μg/mL) human cancer cell lines. The antiproliferative potential of the above-mentioned extracts was also confirmed by automatic time-lapse fluorescent image capture (Figure 1). Although these extracts do not induce significant cell death at a tested concentration of 100 μg/mL (Figure 2a,b), *A*. *transsilvanica* extracts (leaves and roots) significantly inhibit the proliferation of Caco-2 and HT29 cell lines treated for 72 h as compared to non-treated cells (Figure 2c,d). *A*. *transsilvanica* is an endemic species from Romania, and regrettably, its chemical composition has not been previously characterized. However, other authors have described that saponins are the most abundant compounds found in the *Anemone* species, along with significant amounts of ranunculin, anemonin, and protoanemonin [48,49,50]. Han et al. [51] suggested that triterpenoid saponins isolated from *Anemone flaccida* are involved in the suppression of hepatocellular carcinoma through multiple signaling cascades related to tumorigenesis and tumor metabolism, demonstrating high potential applications in cancer therapy. Furthermore, there is evidence that Raddeanin A, an oleanane-type triterpenoid saponin described as the main bioactive constituent extracted from the root of *Anemone raddeana* [52], inhibits proliferation and induces apoptosis in various human tumor cells, including gastric cancer cells, hepatocellular carcinoma cells, and non-small-cell lung carcinoma cells [53,54,55,56]. Further research indicates that Raddeanin A is an effective inhibitor of breast cancer-induced osteolysis [57]. Accordingly, it is tempting to assume that triterpene saponins are involved in *A*. *transsilvanica* bioactivity.

On the other hand, *A*. *moldavicum* and *C*. *palustris* extracts (100 μg/mL) were demonstrated to be the most effective in inducing cell death in both adenocarcinoma cell lines tested within the first 5 to 10 h of extract exposure (Figure 2a,b). As far as the results of the MTT assay, the IC_50_ values calculated for extracts obtained from *A*. *moldavicum* and *C*. *palustris* were 160.7 ± 7.5 and 85.8 ± 7.3 μg/mL for Caco-2, and 77.5 ± 10.4 and 148.8 ± 0.5 μg/mL for HT29 cells, respectively. Furthermore, *A*. *moldavicum* and *C*. *palustris* extracts demonstrated relatively low toxicity against a normal epithelial cell line with an IC_50_ of 291.2 ± 7.2 and 277.4 ± 16.9 μg/mL, respectively.

Antiproliferative activity was also detected for *R*. *serpens* subsp. *nemorosus* extract against tested Caco-2 (IC_50_ = 83.3 ± 4.6 μg/mL) and HT29 (IC_50_ = 67.4 ± 1.8 μg/mL) cells. Moreover, based on the results of cell death assay, *R*. *serpens* subsp. *nemorosus* extract exposure significantly induced cell death in Caco-2 cells (Figure 2a). The root extracts of *H*. *nobilis* exhibited the maximum selective cytotoxicity toward Caco-2 adenocarcinoma cells, with an IC_50_ of 46.9 ± 5.9 μg/mL and a selectivity index at 7.1 in the MTT assay. The remaining extracts exhibited weak antiproliferative activities (IC_50_ ranging from 94.1 to 387.7 μg/mL) or were ineffective (IC_50_ > 512 μg/mL). Toxicity assessment in normal cells revealed that extracts of *A*. *toxicum* (roots), *A*. *variegatum*, *A*. *vulparia*, *R*. *platanifolius*, *R*. *polyanthemos*, *R*. *repens*, and *T*. *europaeus* did not affect the growth of the FHs 74Int cell line at a concentration of 512 μg/mL. The remaining tested extracts demonstrated low toxicity with IC_50_ ranging from 152.9 to 463.6 μg/mL. Although promising anti-cancer activities have been already reported for several genera of the family including *Aconitum*, *Anemone*, and *Ranunculus* [11]; to the best of our knowledge, this is the first report on the antiproliferative activity of *A*. *moldavicum*, *A*. *transsilvanica*, and *R*. *serpens* subsp. *nemorosus*.

The data obtained in previous studies indicate that several *Aconitum* alkaloids (e.g., aconitine, lappaconitine, and taipeinine) exhibit anti-cancer activities [58,59]. Furthermore, diterpenoid alkaloids (e.g., C18-, C19-, C20-, and bis-diterpenoid alkaloid) isolated from *Aconitum* species and *Delphinium* plants are described as the most promising natural compounds for cancer treatment [12]. However, we should be aware that these compounds serve as a double-edged sword because they could be potentially toxic while having beneficial medicinal effects. Although there is increasing evidence that has shown the benefits of using *Aconitum* and *Delphinium* herbs in the treatment of various illnesses, it is of high importance to point out that these plants have a high toxicity level in the raw form and are marked as “very poisonous that must be used with extreme care” [60]. Nevertheless, further analysis should be carried out to determine the chemical composition of extracts and to ultimately assign the active compound from the extract mixture.

### 2.3. Antileishmanial Effect

The Ranunculaceae crude extracts were tested for their in vitro effects on extracellular and intracellular forms of *Leishmania infantum*. The parasitic disease leishmaniasis is a major health problem worldwide affecting millions of people, especially in developing nations [61]. *L*. *infantum* causes visceral leishmaniasis, the most severe form, involving infections of the liver, spleen, and bone marrow and which is associated with high fatality. Current medications are having considerable side effects and are expensive to access. Furthermore, emerging resistant strains limit the application of current treatments in endemic areas. Therefore, the development of effective and affordable chemotherapeutic agents offering novel cures for leishmaniasis is an urgent research priority [62].

**Figure 3:**
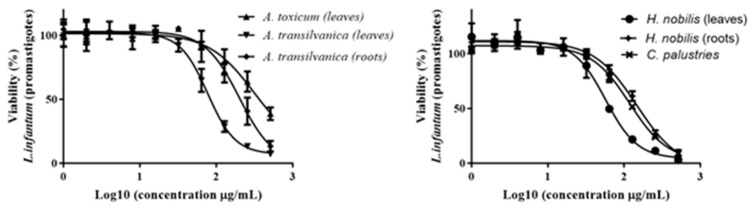
Dose-response (IC_50_) of *A*. *toxicum*, *A*. *transilvanica* (leaves and roots), *H*. *nobilis* (herb and roots), and *C*. *palustries* extracts against *L*. *infantum* promastigotes.

**Figure 4:**
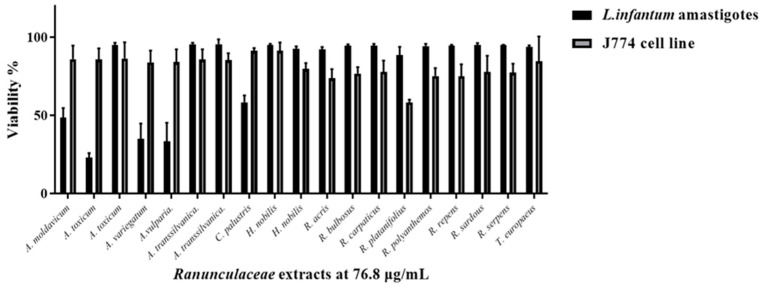
Single-point parasite rescue assay for *L. infantum* intracellular amastigotes. A bar graph shows antiparasitic activity of 19 Ranunculaceae ethanolic extracts at a concentration of 76.8 μg/mL against *L*. *infantum* amastigotes and cytotoxicity against J774 macrophage host cells, expressed as % cell viability +/− standard deviation.

Among all tested extracts, *A*. *transsilvanica* (leaves) and *H*. *nobilis* (herb and roots) extracts exhibit the highest potency against *L*. *infantum* promastigotes with IC_50_ = 18.6 ± 6.5, 19.5 ± 6.4 and 22.1 ± 5.8 μg/mL, respectively (Figure 3). Additionally, *C*. *palustris* (IC_50_ = 30.7 ± 1.8 μg/mL), *A*. *toxicum* (herb extract IC_50_ = 34.7 ± 20.7 μg/mL), *A*. *transsilvanica* (roots IC_50_ = 41.0 ± 24.0 μg/mL), *R. platanifolius* (IC_50_ = 41 ± 11.1 μg/mL), and *R*. *repens* (IC_50_ = 47 ± 11.1 μg/mL) extracts demonstrated significant antileishmanial activity against the promastigote form of *L*. *infantum* (Figure 3). Furthermore, the selectivity index of the above-mentioned extracts ranged from 7.9 to 23.8, indicating a relatively large window between cytotoxicity and antiparasitic activity. The remaining extracts exhibit an IC_50_ value ranging from 55 to 310 μg/mL. IC_50_ values obtained after 72 h of extract exposure for *L*. *infantum* promastigote form are summarized in Table 2. Due to the intracellular nature of *Leishmania* infection, effective chemical compounds must be able to eliminate the parasite inside the host cell. Therefore, other than sufficient selectivity, which is an essential feature of drug candidates, anti-leishmanial compounds should possess the ability to penetrate the mammalian host cell to clear the intracellular form of the parasite. Evaluating all the extracts at a concentration of 76.8 µg/mL, we identified two *Aconitum* species (*A*. *toxicum*, and *A*. *vulparia*) extracts successfully limiting intracellular parasite growth without having a significant toxic effect on the murine macrophage host cell J774 (Figure 4).

**Figure 5:**
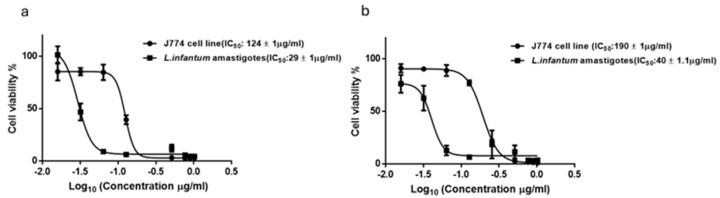
Dose-response analysis (IC_50_) of *A*. *vulparia* (**a**) and *A*. *toxicum* herb (**b**) extracts for *L. infantum* intracellular amastigotes. Derived IC_50_ values against amastigotes and J774 host macrophages for each extract are indicated in the legend (n = 3).

Detailed dose-response analysis (Figure 5a) showed that the *A*. *vulparia* extract was the most effective against the intracellular amastigote form of *L*. *infantum* with an IC_50_ of 29 ± 1 µg/mL in the rescue assay and the *A*. *toxicum* extract resulted in an IC_50_ equal to 40 ± 1 µg/mL (Figure 5b). Cytotoxicity for J774 host macrophages, monitored in parallel, revealed selectivity indexes of 4.7 and 4.2, respectively, with IC_50_ of 124 ± 1 µg/mL (*A*. *vulparia*) and IC_50_ of 190 ± 1.1 µg/mL (*A*. *toxicum*) extracts.

These results indicate that the active leishmanicidal compound(s) of these extracts are efficiently entering the host cell. The modest therapeutic window may be optimized through further studies aimed at identifying the exact effective fraction of these extracts. Recent studies have shown that alkaloids and berberine compounds isolated from various plants are important sources of drug candidates against leishmaniasis. Regarding the Ranunculaceae species, there are shreds of evidence that extracts and essential oils isolated from *Nigella sativa* seeds possess in vitro and in vivo antileishmanial activity [63,64,65]. Additionally, diterpenpene alkaloids such as 15,22-O-diacetyl-19-oxo-dihydroatisine, azitine, and isoazitine obtained from *Aconitum*, *Delphinium*, and *Consolida* species, exhibit leishmanicidal activities against the promastigotes form of *L*. *infantum* [20]. Furthermore, several flavonoid derivatives from *Aconitum napellus* subsp. *lusitanicum*, *Consolida oliveriana*, *Delphinium gracile*, and *D*. *staphisagria* exhibit a leishmanicidal effect against promastigote as well as amastigote forms of *L*. *donovani*, *L*. *braziliensis*, and *L*. *infantum* at similar concentrations as the reference drug (Glucantime) [66,67,68]. However, to the best of our knowledge, this is the first report demonstrating the antileishmanial activity of extracts of *A*. *transsilvanica*, *A*. *toxicum*, *A*. *vulparia*, and *H*. *nobilis* against the promastigote form of *L*. *infantum*. Significantly, we demonstrate that *A*. *toxicum* and *A*. *vulparia* extracts have the ability to eliminate the clinically relevant, intracellular form from host macrophages.

Among the extracts studied in the current study, *A. toxicum* and *A.vulparia* extracts are promising candidates for further investigations in animal models of *Leishmania* infection. Furthermore, combinational therapy of these candidates alongside considerably toxic generic drugs like Amphotericine B or Miltefosine could be considered in animal model studies to reduce the required dosage to less toxic levels [69] as well as lowering the risk of developing resistance [70]. Moreover, the possibility of an immunomodulatory effect of these herbal extracts or their derivatives [71], warranting further investigation, could potentially improve drug response by providing activation and dominance of beneficial TH1 (T helper 1) immune responses and activation of M1 type macrophages which support an ideal healing profile for *Leishmania* infection [29,72]. Also notably, the origin of these candidate extracts from the genus *Ranunculus*, widely accepted as traditional wound healers [73], may potentiate the curative effect when applied as topical medications either in the form of cream or as a bioactive compound in wound dressing bandages for cutaneous leishmaniasis [74].

### 2.4. Antitrypanosomal Effect

Extracts were also tested for in vitro potency against *Trypanosoma brucei*, the causative agent of human and animal African trypanosomiasis, also known as sleeping sickness in humans and nagana in animals, exerting a considerable impact on sub-Saharan African economics and public health. The parasite is transmitted by the blood-feeding tsetse fly vector (*Glossina* sp.), and the resulting infection is usually fatal unless treated. As far as the antitrypanosomal activity of the Ranunculaceae species is considered, there are just a few reports in this direction. Herrmann et al. [75] reported the strong activity of *Coptis chinensis* methanolic extract against *T*. *brucei* with an IC_50_ of 0.4 µg/mL. Another study, by Marin et al. [21], shows that several flavonoid glycosides isolated from *Aconitum napellus* subsp. *lusitanicum*, *Consolida oliveriana*, and *Delphinium gracile* exhibit higher trypanocidal activities than the reference drug benznidazole against the intracellular forms of *Trypanosoma cruzi*.

**Figure 6:**
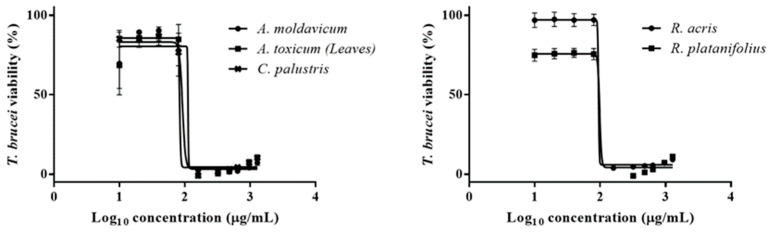
Dose-response analyses (IC_50_) of *A. moldavicum, A. toxicum* (herb), *C. palustries*, *R. acris*, and *R. platanifolius* extracts against bloodstream form of *T. brucei*. Corresponding IC_50s_ are listed in Table 2.

In the current study, the ethanolic extracts showed only moderate or low activity against *T*. *brucei*. Out of all the tested extracts, *A*. *toxicum* and *C*. *palustris* exhibited the lowest IC_50_ values of 88.8 ± 25 and 83 µg/mL. Additionally, *A*. *moldavicum* (IC_50_ = 89.9 ± 1.6 μg/mL), *R*. *acris* (IC_50_ = 91.4 ± 3.7 μg/mL), and *R*. *platanifolius* (IC_50_ = 98.3 ± 0.4 μg/mL) extracts revealed similar antitrypanosomal potential (Figure 6). The remaining extracts displayed an IC_50_ ranging from 102.6 to 269.3 against *T*. *brucei*. To the best of our knowledge, this is the first report concerning the antitrypanosomal effect of all plants tested in the present study. However, further analyses should be carried out to identify the active compound(s) from the extract mixture.

### 2.5. Combined Antioxidant and Antiproliferative Activity

Previously it was reported that the progression of cancer is strongly related to oxidative stress [76]. It is considered that reactive oxygen species (ROS) act as secondary messengers and in overdose can initiate DNA damage (e.g., oxidized DNA bases), which might ultimately lead to carcinogenesis and tumor promotion [77,78]. Currently, many studies combine chemotherapy with certain types of antioxidants (e.g., curcuminoid, β-carotene, glutathione, glutamine, resveratrol, selenium, vitamins C and E) due to their potential to decrease the toxic effect of medication [79,80]. In fact, much attention has been focused on the antioxidative and antiproliferative compounds isolated from various plant species, to provide clues for modern drug discovery [81,82,83]. Taking into account all of the aforementioned, we also assessed extracts for exerting dual antioxidant and anticancer activity. Among all tested plant extracts, combined antioxidant and antiproliferative effects were observed for *A*. *moldavicum* and *C*. *palustris* together with their relatively low toxicity toward FHs 74Int cell lines. Although extracts of *A*. *moldavicum* are locally used to treat cough, neuralgia, and rheumatism, their efficacies have not been scientifically validated. According to Borcsa et al. [84], the chemical composition of *A*. *moldavicum* is mainly characterized by high amounts of diterpenoid and norditerpene alkaloids. Additionally, the presence of certain flavonoids was also reported in various *Aconitum* species [31,36], but studies on the flavonoids content of *A*. *moldavicum* are lacking. Therefore, it remains unclear if alkaloids, flavonoids, or phenolic compounds play a role in the observed combinatory activities of *A*. *moldavicum*. Notably, the results obtained in our research for *C*. *palustris* are in agreement with the findings of Mubashir et al. [85], who reported a broad spectrum of bioactivities for the respective root methanolic extract, including antioxidant and antiproliferative effects.

A favorable role of antioxidant activity has also been recognized for the treatment of parasitic infections, by means of selectively reducing oxidative damage in the host caused by reactive oxygen or nitrogen species [86]. While the generation of the latter radicals is an integral part of the host immune response, several antiprotozoal frontline treatment options rely on nitrodrugs, which function as prodrugs that are selectively activated by radical formation inside the parasite cell. This mode of action is relevant for benznidazole, a frontline treatment for chagas disease [87], nifurtimox, a potent trypanocidal [88,89], and also for nitroimidazoles, which are currently the only treatment option for trichomonaiasis [90]. In this context, future studies could address the suitability of our plant extracts with antioxidant properties for combination therapy with nitrodrugs.

## 3. Materials and Methods

### 3.1. Plant Material

The selection of plant species was based on previously reported data on traditional use for the treatment of diseases likely to be associated with oxidative stress (e.g., chronic inflammation, asthma, arthritis, gout, and rheumatism), cancer, and parasitic diseases (e.g., malaria) [5,6,7]. Plants were collected in Romania from their natural habitats during their main flowering seasons in June–July 2016 and May–August 2017. Except *A. transsilvanica*, which is flowering in early spring, all collected herbs were in the flowering stage during collection. Plants were collected in five mountain areas, namely, Mt. Almajului, Mt. Intorsurii, Mt. Piatra Craiului, Mt. Postavaru, Mt. Stamba, and one city area in Cluj-Napoca. Identification of specimens was performed in the field and the voucher specimens have been deposited in the Herbarium collection (CLA) at the University of Agricultural Sciences and Veterinary Medicine of Cluj-Napoca, Romania. The botanical names, voucher specimen numbers, location and date of plant collection, and traditional uses of plant species are given in Table 1.

### 3.2. Sample Preparation

Plant materials were air-dried at room temperature and finely ground into powder using an electric mill GM100 (Retsch, Haan, Germany) and 5 g of each powdered sample was macerated in 150 mL of 80% ethanol (EtOH) for 24 h at room temperature using a laboratory shaker GFL3005 (GFL, Burgwedel, Germany). Extracts were subsequently filtered and evaporated to dryness using a rotary evaporator R-200 (Buchi, Flawil, Switzerland) in vacuo at 40 °C. Dried residues were dissolved in 100% dimethylsulphoxide (DMSO) to obtain a concentration of 51.2 mg/mL stock solution of each extract, which was stored at –80 °C until tested. Dry residue extraction yield was calculated as follows: extraction yield % = weight of the dry extract after evaporation/weight of the dry plant material used for extraction × 100%. Dried residue extraction yields (%) are shown in Table 2.

### 3.3. Chemicals and Reagents

The following chemicals and reagents, purchased from Sigma–Aldrich (Prague, Czech Republic), were used in this study: 2,2-azobis(2-methylpropionamidine) dihydrochloride (AAPH), 2,2-diphenyl-1-picrylhydrazyl (DPPH), 6-hydroxy-2,5,7,8-tetramethylchromane-2-carboxylic acid (Trolox), Dulbecco’s modified Eagle’s medium (DMEM), human epidermal growth factor, fetal calf serum (FCS), fluorescein (FL), non-essential amino acids, penicillin-streptomycin solutions, RPMI medium, thiazolyl blue tetrazolium bromide (MTT), and vinorelbine. DMSO, EtOH, and methanol were purchased from Penta (Prague, Czech Republic). Hybri-Care Medium 46-X was purchased from American Type Culture Collection (ATCC; Rockville, MD, USA). Medium 199 (M199), Minimum Essential Media (MEM), fetal bovine serum (FCS), BME vitamins, Phorbol 12-myristate 13-acetate (PMA), and SYTOX Green death cell stain (S34860) were purchased from Thermo Fisher Scientific (Prague, Czech Republic). Amikin was prepared from Medopharm (Kodambakkam Chennai, Tamilnadu, India) and resazurin was obtained from Chem Cruz (Dallas, TX, USA).

### 3.4. Cell Cultures

Human colon adenocarcinoma cell lines Caco-2 and HT29 (ATCC, Rockville, MD, USA) were maintained in DMEM supplemented with FCS (10%), 1 mM sodium bicarbonate, 1 mM sodium pyruvate, non-essential amino acids (1%), and penicillin-streptomycin solution (100 U/mL). Normal small intestinal cell line FHs 74Int (ATCC, Rockville, MD, USA) was maintained in Hybri-Care medium 46-X supplemented with 10% FCS, penicillin-streptomycin solution (100 U/mL) and 30 ng/mL human epidermal growth factor. Murine macrophage cell line (J774) was cultivated in vitro in RPMI supplemented with 10% FCS, 1% MEM and penicillin-streptomycin solution (100 U/mL), and PMA (50 µg/mL). Cell cultures were incubated in a 5% CO_2_ atmosphere at 37 °C using an MCO 170AIC-PE CO_2_ incubator (Panasonic Corporation, Osaka, Japan).

### 3.5. Parasite Strains and Culture

*Leishmania infantum* (MHOM/BR/76/M4192) promastigotes were cultivated in vitro in M199 media supplemented with NaHCO_3_ (1 mM), BME Vitamins (1%), human urine (0.1%; collected from a healthy person), Amikin (0.1%), and 10% FCS. Addition of human urine to leishmania culture is popular for improving cell growth and has become a widely used media component [91,92]. The culture was kept in Roux flasks with a surface area of 25 cm^2^ at 27 °C in an air atmosphere using an MCO 170AIC-PE CO_2_ incubator (PHCbi, Tokyo, Japan). The bloodstream form of *Trypanosoma brucei brucei* (*T*. *brucei*), derived from Lister strain 427 (Molteno Institute Trypanosomal antigen type (MITat) 1.2) were cultured in HMI-9 complete medium (HMI-9 supplemented with 10% FCS, 100 U/mL penicillin, and 100 U/mL streptomycin at 37 °C with 5% CO_2_ in a humid atmosphere) [93].

### 3.6. Antioxidant Activity

#### 3.6.1. Oxygen Radical Absorbance Capacity (ORAC) Assay

The ORAC method previously described by Ou et al. in 2001 [94] and slightly modified by Tauchen et al. [95] and Rondevaldova et al. [96] was used for the evaluation of the ability of the extracts to protect FL from AAPH-induced damage. Firstly, the serial dilutions (final concentration range: 256 to 1 μg/mL) of each extract were prepared in phosphate buffer in black absorbance 96-well microtiter plates using the automated pipetting platform Freedom EVO 100 (Tecan, Mannedorf, Switzerland). Blank and positive control wells received phosphate buffer and synthetic antioxidant (Trolox) dilution, respectively. After, the addition of 150 μL FL (480 μM) prepared in 75 mM phosphate buffer (pH 7.0) to each of the well plates was incubated at 37 °C for 10 min. Subsequently, the reaction was started by applying 25 μL AAPH (153 mM). The plates were incubated for another 90 min at 37 °C. Immediately, the fluorescence changes were measured using a Cytation 3 microplate reader, using Gen5 software, version 3.11 (BioTek, Winooski, VT, USA) with λ_exc_: 485 nm and λ_em_: 528 nm. The fluorescence signals were used to monitor the rate of FL quenching.

#### 3.6.2. DPPH Radical Scavenging Assay

The ability of extracts to neutralize DPPH was determined using a slightly modified method previously described by Sharma and Bhat [97]. Serial dilutions of each sample were prepared in absolute methanol (100 μL) in 96-well microtiter plates using the automated pipetting platform Freedom EVO 100 (Tecan, Mannedorf, Switzerland). Blank and positive control wells received methanol and Trolox dilution, respectively. The reaction was started by adding 75 μL of absolute methanol and 25 μL of freshly prepared 1 mM DPPH to each well, creating a range of concentrations from 0.125 to 256 μg/mL. The mixture was kept in the dark at room temperature for 30 min. Absorbance was measured at 517 nm using a Cytation 3 microplate reader, using Gen5 software, version 3.11 (BioTek, Winooski, VT, USA).

### 3.7. Cytotoxicity Activity

#### 3.7.1. Cell Viability Assay

A modified method based on the metabolization of yellow MTT to blue formazan by mitochondrial dehydrogenases in living cells previously described by Mosmann in 1983 [98] was used to test cell viability. Caco-2, HT29, and FHs 74Int cell lines were preincubated in a 96-well plate at a density of 2.5 × 10^3^ (Caco-2 and HT29) and 2.5 × 10^5^ (FHs 74Int) cells per well for 24 h in a humidified atmosphere of 5% CO_2_ in air at 37 °C. After 24 h, the cells were treated with two-fold serial diluted samples (16 to 512 μg/mL) for 72 h. Subsequently, fresh DMEM or Hybri-Care medium containing MTT reagent (1 mg/mL) was added to each well, and plates were incubated for an additional 2 h at 37 °C. The media were then removed, and the intracellular formazan product was dissolved in 100 μL of DMSO. The absorbance was measured at 555 nm using a Tecan Infinite M200 spectrometer (Tecan Group, Mannedorf, Switzerland). Vinorelbine was used as a positive control (at a concentration range of 0.001 to 16 μg/mL).

#### 3.7.2. Cell Death Assay

Cell death and the percent of the proliferation of Caco-2 and HT29 cell lines treated with the most promising extracts were evaluated using the automated brightfield and fluorescent image capture with a Lumascope 720 microscope (Etaluma, Carlsbad, CA, USA). Firstly, human colon adenocarcinoma cell lines were preincubated at 37 °C in 96-well plates at a density of 5 × 10^3^ cells per well for 24 h with 5% CO_2_. Subsequently, cells were treated with the six extracts, namely, *A*. *moldavicum*, *A*. *transsilvanica* (leaves and root extracts), *C*. *palustris*, *H*. *nobilis*, and *R*. *serpens* subsp. *nemorosus* at concentration of 100 μg/mL. The Green dead cell stain SYTOX (15 nM/well) was added together with the extracts to each well and automated brightfield and fluorescent image capture was performed every 2 h at 100× on a LumascopeTM 720. The number of SYTOX positive cells, as well as the percent of proliferation, were quantified using Lumaquant 8.8 software (Etaluma, Carlsbad, CA, USA).

### 3.8. Antiparasitic Activity

#### 3.8.1. Resazurin Assay

The growth inhibitory effects of the extracts against *L*. *infantum* (promastigote form) and *T*. *brucei* (blood stream form) were evaluated using a resazurin-based viability assay. Firstly, each extract was dispensed into a white, tissue culture-treated, 384 microtiter plates (BD Falcon, Franklin Lakes, NJ, USA) using an ECHO 550 acoustic dispenser (Labcyte, San Jose, CA, USA) to prepare for a final concentration range of 1 to 1024 μg/mL. Subsequently, *L*. *infantum* and *T*. *brucei* cells in medium were added at a density of 5 × 10^5^ and 10^3^ cells/well, respectively, to a volume of 100 μL/well. The plates were incubated at 27 °C (*L*. *infantum*) or 37 °C in a humid, 5% CO_2_ atmosphere (*T*. *brucei*) for 72 h. Then, resazurin solution (500 µM in PBS) was added in the ratio of 1:10 (vol/vol) to each well and incubated overnight in order to assess the viability of *L*. *infantum* and *T*. *brucei*. The fluorescence intensity of the samples was measured with a plate reader (Biotek, Winooski, VT, USA) at λ_exc_: 550 nm and λ_em_: 590 nm. IC_50_ values were calculated from dose-response curves based on non-linear regression in GrapPad Prism (San Diego, CA, USA). Amphotericin (*L*. *infantum*) and the phenoxybenzoxaborole [99] AN3057 (*T*. *brucei*) were used as a positive control.

#### 3.8.2. Parasite Rescue Assay

Extracts were evaluated against intracellular amastigote stage of *L. infantum* using a parasite rescue assay described previously by Zahedifard et al. [100]. First, murine macrophage cells (J774) were seeded (5 × 10^4^ cell/well) for 24 h to differentiate. Afterward, the cells were washed with warm (37 °C) serum-free RPMI. Stationary phase promastigotes of *L*. *infantum* were diluted in RPMI 2% FCS and added in the ratio of 10:1 (parasite: cell) (5 × 10^5^ cell/well). After 24 h incubation, cells were washed five times with serum-free RPMI to remove extracellular parasites and exposed to extracts at concentrations (ranging from 16 to 1024 μg/mL). The extract dilutions were prepared in RPMI (2% FCS) using an ECHO 550 acoustic dispenser (Labcyte, San Jose, CA, USA). After 48 h, three washing steps were performed, and afterward, cells were lysed with 20 µL of 0.05% SDS in RPMI for 30 s and liberated parasites incubated at 26 °C with M199 for another 72 h. Lastly, the viability of these cells was measured based on resazurin assay as described above. Percent of viability was normalized using non-treated and no cell controls [101].

### 3.9. Statistical Analysis

All tests were carried out in triplicate. ORAC, DPPH, and MTT assays were performed in three independent experiments, while resazurin and parasite rescue assays were repeated twice (in biological duplicate, each in technical triplicate). Results were expressed as an IC_50_ in μg/mL for the above-mentioned assays. In ORAC and DPPH, the concentration of the extract ((IC_50_ (μg/mL))required to inhibit 50% of the radical was calculated using linear regression analysis. For MTT, resazurin, and parasite rescue assays, the IC_50_ (μg/mL) was determined as the concentration of the respective extract required to reduce 50% of cell viability when compared to the non-treated control. The selectivity of anticancer and antiparasitic extracts was determined in order to evaluate their safety. Thus, the selectivity index (SI) was calculated as the ratio of the geometric mean of IC_50_ values of normal small intestinal cell line FHs 74Int to IC_50_ values calculated for colon adenocarcinoma cell lines Caco-2 and HT29 cells or the IC_50_ values calculated for *L*. *infantum* and *T*. *brucei*, respectively. Higher SI is thus indicative of a safer extract. Statistical analysis was performed in Magellan software, version 7.2 (Tecan Group, Mannedorf, Switzerland) GraphPad Prism 6.0 software (San Diego, CA, USA) and Microsoft Office Excel 2016 (Microsoft, Redmond, WA, USA).

## 4. Conclusions

In summary, the main objective of this study was to determine the in vitro antioxidant, anticancer, and antiparasitic potential of ethanolic extracts obtained from 16 Ranunculaceae species. Our studies revealed a significant antioxidant potential of extracts of *A*. *toxicum*, comparable to the prototypic antioxidant Trolox. Furthermore, *A*. *transsilvanica* and *R*. *serpens* subsp. *nemorosus* extracts produced a selective antiproliferative effect against carcinogenic and non-carcinogenic cell lines. The results also showed that *A*. *moldavicum* and *C*. *palustris* extracts exhibit strong cytotoxicity potential against adenocarcinoma cell lines combined with free radical scavenging ability. A further interesting finding of the study is the selective cytotoxic effect of *A*. *vulparia* and *A*. *toxicum* extracts against the intracellular form of *L*. *infantum*. It remains a major challenge to target intracellular pathogens, and our results indicate that the active leishmanicidal compounds of these extracts are efficiently entering the host cell. Ultimately, the above-mentioned plant extracts can be considered as prospective materials for the further development of novel plant-based anti-leishmanicidal, antioxidant, and/or antiproliferative compounds. However, further research should be carried out with the aim of isolating the bioactive compounds responsible for their biological effects. 

## Figures and Tables

**Table 1:** Ethnobotanical data on tested Ranunculaceae medicinal plants.

Plant Species	Date of Collection	VSN	Plant Origin	Collected Site	Ethnomedicinal Uses *
*Aconitum moldavicum* Hacq.	June 2016	CLA30049	Mt. Stamba	46°12’50.8″ N, 22°51′35.4″ E	Treatment of arthritis, gout, and rheumatism (herb and root) [5,6,7]
*Aconitum toxicum* Rchb.	August 2017	CLA30063	Mt. Piatra Craiului	45°33′12.6″ N, 25°25′27.0″ E	Treatment of skin tumors (herb and root) [5,6,7]
*Aconitum variegatum* L.	June 2016	CLA30048	Mt. Stamba	46°12′50.8″ N, 22°51′35.4″ E	Analgesic and antirheumatic properties (herb) [5,6,7]
*Aconitum vulparia* Rchb.	July 2016	CLA30046	Mt. Postavaru	45°34′56.1″ N, 25°33′21.7″ E	Treatment of chronic skin disorders and rheumatism (herb) [5,6,7]
*Anemone transsilvanica* Fuss.	July 2016	CLA30047	Mt. Postavaru	45°34′56.1″ N, 25°33′21.7″ E	Treatment of bronchiti,. cataract, and rheumatic gout (root) [5,6,7]
*Caltha palustris* L.	May 2017	CLA30064	Mt. Intorsurii	45°37′53.4″ N, 26°08′36.7″ E	Treatment of arthritis, gout, rheumatism, anemia, and parasitic diseases (herb) [5,6,7]
*Hepatica nobilis* Mill.	May 2017	CLA30065	Mt. Almajului	44°40′46.1″ N, 21°42′28.4″ E	Treatment of various inflammation, rheumatism, and microbial infections (herb and root) [5,6,7]
*Ranunculus acris* L.	June 2016	CLA30042	Mt. Stamba	46°12′50.8″ N, 22°51′35.4″ E	Treatment of asthma, bronchitis, malaria, and rheumatism (herb) [5,6,7]
*Ranunculus bulbosus* L.	June 2016	CLA30050	Mt. Stamba	46°12′50.8″ N, 22°51′35.4″ E	Treatment for arthritis, gout, and neuralgia pains (herb) [5,6,7]
*Ranunculus carpaticus* Herbich	June 2017	CLA30044	Mt. Postavaru	45°34′56.1″ N, 25°33′21.7″ E	Treatment of various inflammation and skin infections (herb) [5,6,7]
*Ranunculus platanifolius* L.	June 2017	CLA30040	Mt. Postavaru	45°34′56.1″ N, 25°33′21.7″ E	Treatment of rheumatism (herb) [5,6,7]
*Ranunculus polyanthemos* L.	June 2016	CLA30051	Mt. Stamba	46°12′50.8″ N, 22°51′35.4″ E	Treatment of rheumatism, gastric, and duodenal ulcers (herb) [5,6,7]
*Ranunculus repens* L.	June 2016	CLA30045	Mt. Stamba	46°12′50.8″ N, 22°51′35.4″ E	Treatment of malaria and rheumatism (herb) [5,6,7]
*Ranunculus sardous* Crantz	July 2016	CLA30067	Cluj-Napoca	46°46′26.1″ N, 23°34′55.2″ E	Treatment of various inflammation (herb) [5,6,7]
*Ranunculus serpens* subsp. *nemorosus* L.	June 2017	CLA30043	Mt. Postavaru	45°34′56.1″ N, 25°33′21.7″ E	Treatment of various inflammation and rheumatism (herb) [5,6,7]
*Trollius europaeus* L.	June 2017	CLA30066	Mt. Postavaru	45°34′56.1″ N, 25°33′21.7″ E	Treatment of various types of inflammation and bacterial infections (herb) [5,6,7]

VSN: voucher specimen number; ^*^ References.

**Table 2:** In vitro antioxidant, antiproliferative, and antiparasitic activity of Ranunculaceae crude extracts.

Species	PPT	EY (%)	Assay/Cell Line/Parasite/IC_50_ (µg/mL) ^1^										
			DPPH	ORAC	MTT					Resazurin Assay			
					Caco-2 ^2^	SI	HT29 ^2^	SI	FHs 74Int ^3^	*L. infantum* (Promastigote)	SI	*T. brucei*	SI
*A. moldavicum*	H	5.6	104.0 ± 2.6	23.1 ± 4.6	160.7 ± 7.5	1.8	77.5 ± 10.4	3.8	291.2 ± 7.2	115.5 ± 7.2	2.5	89.9 ± 1.6	3.2
*A. toxicum*	H	5.8	92.6 ± 16.6	18.7 ± 6.6	292.9 ± 16.6	0.9	228.2 ± 15.7	1.2	274.9 ± 26.2	34.7 ± 20.7	7.9	88.8 ± 25	3.1
	R	10.7	192.2 ± 18.2	45.7 ± 11	245.2 ± 10.3	2.1	>512	1.0	>512	230.0 ± 87.4	2.2	119.8 ± 11	4.3
*A. variegatum*	H	4.2	119.8 ± 15.0	26.4 ± 7.7	144.3 ± 7.5	3.5	330.7 ± 17.9	1.5	>512	310.0 ± 12.2	1.7	106.1 ± 21	4.8
*A. vulparia*	H	5.5	137 ± 6.6	56.4 ± 2.5	249.9 ± 0.8	2.0	184.5 ± 2.1	2.8	>512	221.3 ± 14.1	2.3	113.4 ± 36	4.5
*A. transsilvanica*	L	11.5	179.6 ± 12.9	35.7 ± 14.7	46.9 ± 5.9	3.3	86.5 ± 4.5	1.8	154.8 ± 17.2	18.6 ± 6.5	8.3	102.6 ± 14	1.5
	R	7.4	>256	212.0 ± 22.9	65.8 ± 2.6	3.9	70.2 ± 9.4	3.7	259.6 ± 19.1	41.0 ± 24.0	6.3	110.9 ± 1.2	2.3
*C. palustris*	H	7.3	98.5 ± 14.7	35.9 ± 1.6	85.8 ± 7.3	3.2	148.8 ± 0.5	1.9	277.4 ± 16.9	30.7 ± 1.8	9.0	83.0 ± 0.0	3.3
*H. nobilis*	H	6.4	116.5 ± 21.5	46.2 ± 10.1	124.1 ± 1.1	3.7	335.8 ± 19.3	1.4	463.6 ± 12.0	19.5 ± 6.4	23.8	122.4 ± 24	3.8
	R	6.9	>256	238.6 ± 14.1	46.9 ± 4.4	7.1	133.0 ± 11.1	2.5	335.3 ± 10.6	22.1 ± 5.8	15.2	161.7 ± 1.9	2.1
*R. acris*	H	5.7	161.7 ± 24.4	36.7 ± 8.8	>512	0.9	205.2 ± 15.4	2.2	450.3 ± 12.1	55.3 ± 1.9	8.2	91.4 ± 3.7	4.9
*R. bulbosus*	H	7.2	166.9 ± 10.1	62.9 ± 11.1	152.3 ± 6.3	1.9	221.9 ± 5.6	1.3	285.1 ± 6.3	139 ± 25.4	2.1	170.0 ± 9.9	1.7
*R. carpaticus*	H	6.9	207.9 ± 2.0	69.3 ± 9.1	277.6 ± 9.6	1.1	387.7 ± 0.5	0.8	318.9 ± 19.8	93.6 ± 11.2	3.4	171.6 ± 13	1.9
*R. platanifolius*	H	9.5	>256	41.5 ± 6.9	242.5 ± 6.8	2.1	253.2 ± 0.4	2.0	>512	41.4 ± 11.1	12.5	98.3 ± 0.4	5.2
*R. polyanthemos*	H	6.0	157.8 ± 17.3	99.1 ± 6.9	318.2 ± 5.8	1.6	230.7 ± 9.3	2.2	>512	84.0 ± 12.1	6.1	158.6 ± 22	3.2
*R. repens*	H	8.2	190.1 ± 17.9	41.6 ± 6.4	227.6 ± 5.9	2.2	295.2 ± 18.0	1.7	>512	47.9 ± 11.1	10.7	128.7 ± 60.3	4.0
*R. sardous*	H	3.1	182.7 ± 25.5	62.0 ± 6.5	94.1 ± 4.4	3.8	347.9 ± 7.3	1.0	353.5 ± 21.9	131.0 ± 23.7	2.7	132.0 ± 55	2.7
*R. serpens* subsp. *nemorosus*	H	4.6	240.5 ± 12.8	47.7 ± 8.5	83.3 ± 4.6	1.8	67.4 ± 1.8	2.3	152.9 ± 4.6	87.4 ± 13.0	1.8	138.2 ± 51.1	1.1
*T. europaeus*	H	5.2	174.6 ± 40.0	38.9 ± 8.9	213.9 ± 32.7	2.4	>512	-	>512	108.2 ± 1.1	4.7	269.3 ± 74.0	1.9
DMSO	-	-	-	-	>1024	-	>1024	-	>1024	>512	-	830.2 ± 42.2	-
Trolox ^4^	-	-	14.7 ± 3.5	22.4 ± 7.3	-	-	-	-	-	-	-	-	-
Vinorelbine ^4^	-	-	-	-	0.03 ± 0.02	-	-	-	0.45 ± 0.12	-	-	-	-
Amphotericin ^4^	-	-	-	-	-	-	-	-	-	0.184 ± 0.1	-	-	-
AN-3057 ^4^	-	-	-	-	-	-	-	-	-	-	-	0.019	-

H: herb, defined as a stem with leaves and flowers; DMSO: dimethyl sulfoxide; DPPH: 1-diphenyl-2-picrylhydrazyl assay; EY: extraction yield; IC_50_: half-maximal inhibitory concentration; MTT: 3-[4,5-dimethylthiazol-2-yl]-2,5 diphenyl tetrazolium bromide; ORAC: oxygen radical absorbance capacity assay; PPT: plant part tested; SI: selectivity index (calculated as the ratio of geometric mean of IC_50_ values’ normal cells epithelial FHs 74Int to IC_50_ values calculated for cancer cell Caco-2 and HT29 cells or IC_50_ values calculated for *L*. *infantum* and *T*. *brucei*, respectively); R: roots; L: leaves. ^1^ IC_50_ expressed as mean value ± standard deviation, DPPH, ORAC, and MTT assays have been carried out in three independent experiments while resazurin assay for *L*. *infantum* (promastigotes form) and *T*. *brucei* were performed twice in technical triplicate; ^2^ human colon adenocarcinoma cell line; ^3^ normal small intestine cell line; ^4^ positive control.

## Data Availability

Data underlying this article are available in the article.
